# Biochemical signatures of skin α-synuclein in synucleinopathies revealed by RT-QuIC assay end-product analysis

**DOI:** 10.1007/s00401-025-02973-5

**Published:** 2026-01-12

**Authors:** Maria Gerasimenko, Hancun Yi, Tricia Gilliland, Yijia Chen, Zerui Wang, Wen-Quan Zou

**Affiliations:** 1 Department of Pathology, Case Western Reserve University, Cleveland, OH 44106 USA; 2 Institute of Neurology, Jiangxi Academy of Clinical Medical Sciences and The First Affiliated Hospital, Jiangxi Medical College, Nanchang University, Nanchang, 330200 Jiangxi China

**Keywords:** Synucleinopathy, RT-QuIC, Skin, α-Synuclein, Parkinson’s disease, Dementia of Lewy body, Multiple system atrophy

## Abstract

Synucleinopathies, including Parkinson’s disease (PD), dementia with Lewy bodies (DLB), and multiple system atrophy (MSA), share pathological accumulation of misfolded α-synuclein (αSyn) in the brain and overlapping clinical features, complicating accurate diagnosis with current methods. In this study, we utilized a real-time quaking-induced conversion (RT-QuIC) assay to demonstrate that autopsied skin samples from PD, DLB, and MSA patients (but not non-synucleinopathy controls) seed aggregation of recombinant αSyn. While RT-QuIC generated similarly positive fluorescence kinetic curves across synucleinopathies, biochemical and morphological analyses of RT-QuIC end products revealed distinct properties in the resulting αSyn aggregates. Notably, αSyn aggregates from DLB samples exhibited the highest resistance to proteinase K digestion, whereas MSA-derived aggregates showed the least aggregated bands on Western blots. Transmission electron microscopy revealed significant differences in length, width, and volume of skin αSyn fibrils of RT-QuIC end products from different synucleinopathies. These findings provide critical insights into disease-specific αSyn structural characteristics and suggest new strategies to improve diagnostic discrimination.

**Supplementary Information:**

The online version contains supplementary material available at 10.1007/s00401-025-02973-5.

## Introduction

Synucleinopathies are a group of neurodegenerative disorders defined by the abnormal aggregation and deposition of α-synuclein (αSyn) protein in the brain, including Parkinson’s disease (PD), dementia with Lewy bodies (DLB), and multiple system atrophy (MSA). Despite extensive clinical research, accurately diagnosing these diseases remains difficult due to overlapping symptoms, variable disease progression, and the lack of precise biomarkers. At the molecular level, synucleinopathies share a common feature—the misfolding of αSyn from its native, unfolded form into β-sheet-rich amyloid fibrils. These fibrils aggregate into intracellular structures: Lewy bodies in PD and DLB, and glial cytoplasmic inclusions in MSA. Each disorder shows distinctive patterns and cell types affected [6, 8, 16, 17, 19, 31]. Increasing evidence suggests that αSyn aggregates have prion-like characteristics, where misfolded forms self-propagate and spread through neural networks, potentially influencing disease progression and clinical variability [13–15, 20, 25, 29]. This strain-like behavior of αSyn aggregates has been proposed to underlie the heterogeneity in clinical manifestations across synucleinopathies.

Recent technological advances in seed amplification assays (SAAs) in vitro, particularly real-time quaking-induced conversion (RT-QuIC), have enabled the ultrasensitive detection of minute quantities of αSyn seeds in a range of bio-specimens, including cerebrospinal fluid, skin tissues, submandibular glands, and olfactory mucosa [3, 4, 7, 10, 12, 18, 22, 27]. By exploiting the self-templating property of αSyn aggregates, RT-QuIC facilitates the amplification of misfolded aggregates in vitro using recombinant αSyn (rec-αSyn) as substrate, yielding a kinetic fluorescence signal correlated with aggregate formation [24]. While RT-QuIC has shown high diagnostic accuracy for differentiating synucleinopathies from non-synucleinopathy controls, its capacity to discriminate among PD, DLB, and MSA remains limited, particularly when using peripheral tissues such as skin. More data suggest that αSyn strains from the brain of patients with different synucleinopathies may differ in their structural architecture, biochemical stability, and proteolytic resistance, which could potentially allow for improved differentiation among synucleinopathies [5, 16, 23, 31]. However, detailed characterization of RT-QuIC end products has not been systemically conducted yet, and the extent to which disease-specific structural information is retained in amplified fibrils is unclear. Understanding these strain-specific differences is essential for refining diagnostic accuracy and enhancing our knowledge of disease mechanisms and progression.

In this study, we systemically characterize the biophysical and biochemical properties of αSyn RT-QuIC end products of skin samples from patients with synucleinopathies, including PD, DLB, and MSA. Our examinations include PK resistance conformational stability, sucrose density gradient fractionation, and ultrastructural imaging. Our findings provide new insights into the structural and morphological diversity of αSyn aggregates, underscoring their diagnostic value and highlighting the biological relevance of strain diversity in synucleinopathies.

## Materials and methods

### Source of skin and brain samples

This study utilized autopsy scalp skin samples from 130 subjects, comprising 87 patients with α-synucleinopathies and 43 non-neurodegenerative controls (Table 1). They were procured through the Arizona Study of Aging and Neurodegenerative Disorders/Brain and Body Donation Program (AZSAND/BBDP, Sun City, Arizona, USA). The brain tissue cohort included samples from a subset of the same skin donors (*n* = 17) of ASAND/BBDP as well as cases obtained from the National Prion Disease Pathology Surveillance Center (*n* = 17; 7 PD and 10 non-neurological controls, Cleveland, Ohio, USA). Diagnosis for all cases was confirmed by standard neuropathological examination.

**Table 1:** Demographic and neuropathological characteristics of cases across diagnostic groups

Neuropathological diagnosis	Number of cases	Sex (male/total)	Age (years, mean, SD)	Disease duration (years, mean, SD)	Braak score	Unified LB stage
PD	40	25/40	81.25, 7.994	11.50, 7.87	2 (*n* = 2), 3 (*n* = 19), 4 (*n* = 15), 5 (*n* = 4)	2a (*n* = 4), 2b (*n* = 3), 3 (*n* = 17), 4 (*n* = 16)
DLB	36	21/36	79.92, 8.47	8.41, 2.07	1 (*n* = 1), 2 (*n* = 8), 3 (*n* = 13), 4 (*n* = 9), 5 (*n* = 5)	2b (*n* = 1), 3 (*n* = 6), 4 (*n* = 10)
MSA	11	7/11	76.18, 8.04	6.38, 2.39 (3 cases non-applicable)	1 (*n* = 4), 2 (*n* = 7)	0. No Lewy bodies
NNC	43	27/43	79.44, 14.19	0	1 (*n* = 16), 2 (*n* = 12), 3 (*n* = 15)	0. No Lewy bodies

*PD* Parkinson’s disease, *DLB* dementia with Lewy bodies, *MSA* multiple system atrophy, *NC* neurologically normal controls, *SD* standard deviation, *MoCA* Montreal Cognitive Assessment

*Braak score* staging of neurofibrillary tangle pathology according to Braak and Braak criteria (1–6), *Unified LB Stage* unified Lewy body staging system reflecting the distribution and severity of α-synuclein pathology (stages 1–4), *H&Y* Hoehn and Yahr stage (for disease severity, not listed in the table but relevant to text), *NA* not applicable

### Reagents and antibodies

All chemicals and reagents were obtained from commercial suppliers: Proteinase K (PK) and guanidine hydrochloride (GdnHCl) from Sigma-Aldrich (St. Louis, Missouri, USA); and enhanced chemi-luminescence detection reagents (ECL Plus) from Amersham Pharmacia Biotech (Piscataway, New Jersey, USA). For immuno-detection, the following primary antibodies were used: a mouse monoclonal antibody against αSyn (10D2, Sigma-Aldrich, USA) targeting the 118–127 amino acid region, and a conformation-specific rabbit monoclonal antibody detecting αSyn aggregates (MJFR 14-6-4-2, Abcam, Cambridge, UK), anti-α-synuclein antibody (rabbit monoclonal, MJFR1, ab138501, Abcam, Cambridge, UK). Secondary antibodies were HRP-conjugated sheep anti-mouse IgG (Sigma-Aldrich, USA), donkey anti-rabbit IgG (Cytiva, Marlborough, Massachusetts, USA), and HRP-conjugated goat anti-rabbit IgG (Zenbio, China).

### Preparation of αSyn protein

Recombinant human wild-type αSyn was expressed and purified as previously described [26], with minor modifications. Briefly, a glycerol stock of *E*. *coli* was used to inoculate 4 mL of Terrific Broth (12 g/L tryptone, 24 g/L yeast extract, 4% [v/v] glycerol, 17 mM KH₂PO₄, 72 mM K₂HPO₄) supplemented with antibiotics including ampicillin and chloramphenicol, which was incubated for 6 h at 37 °C. This starter culture was scaled up to 4 L of Terrific Broth and grown overnight at 37 °C. Cells were harvested by centrifugation (6000×*g*, 10 min, 4 °C). The cell pellet was re-suspended in high-salt buffer (750 mM NaCl, 10 mM Tris pH 7.6, 1 mM EDTA) containing protease inhibitors and lysed by sonication. Following lysis, the lysate was boiled for 15 min and centrifuged (10,000×*g*, 20 min, 4 °C) to remove heat-denatured proteins. The supernatant was dialyzed overnight at 4 °C against a low-salt buffer (10 mM Tris pH 7.6, 50 mM NaCl, 1 mM EDTA) and concentrated using a 3 kDa molecular weight cut-off (MWCO) centrifugal filter. The protein was initially purified by size-exclusion chromatography on a Superdex 200 column. Fractions containing αSyn were dialyzed against 10 mM Tris pH 7.6, 25 mM NaCl, 1 mM EDTA and then subjected to anion-exchange chromatography on a HiTrap Q HP column, eluting with a linear salt gradient (25–1000 mM NaCl). αSyn eluted at approximately 300 mM NaCl. The final product was dialyzed against 10 mM Tris pH 7.6, 50 mM NaCl, diluted to 0.5 mg/mL, aliquoted, and stored at –80 °C.

### Skin tissue preparation

Post-mortem skin samples were collected as full-thickness biopsies (30–100 mg, approximately 3–5 mm^2^), comprising primarily the epidermis and dermis. Strict anti-contamination protocols were adhered to during autopsy to prevent cross-contamination between tissue types and donors. For homogenate preparation, samples were processed at a 10% (w/v) concentration in TBS lysis buffer supplemented with 2 mM CaCl₂ and 0.25% (w/v) collagenase A (Roche). The mixture was first digested for 4 h at 37 °C with constant shaking, followed by mechanical disruption using a Mini-Beadbeater.

### RT-QuIC analysis

The real-time quaking-induced conversion (RT-QuIC) assay was performed as previously described [27] with minor modifications. The reaction mixture for brain-derived samples consisted of 50 mM PIPES (pH 7.0), 300 mM NaCl, 7 µM (100 μg/mL) in-house recombinant human wild-type αSyn, 10 µM ThT, and 0.0005% SDS. For skin-derived samples, the mixture contained 150 mM NaCl, 6 µM (87 μg/mL) recombinant αSyn, and 0.00012% SDS, with other components remaining consistent. In both cases, 98 µL of the reaction mix was loaded into a 96-well plate and seeded with 2 µL of the respective homogenate, resulting in final dilutions of 2.5 × 10^−3^ (brain) and 5 × 10^−2^ (skin). The sealed plate was incubated at 42 °C in a BMG FLUOstar Omega plate reader with cycles of 1 min shaking and 1 min rest. ThT fluorescence (excitation 450 ± 10 nm, emission 480 ± 10 nm) was measured every 45 min. All RT-QuIC assays were run for a standardized duration of 56 h, with the ThT fluorescence measured at the final time point recorded as the endpoint fluorescence. Each sample was run in quadruplicate, and the average fluorescence of 4 wells was used for analysis. The threshold was defined as the mean plus five standard deviations of non-PD controls. A sample was considered positive if at least two of its four replicate wells exceeded this threshold.

### Pre-formed fibrils generation

Recombinant human αSyn [4 mg/mL (138 mM)] was aggregated by incubating at 37 °C with constant shaking (1000 rpm) for 7 days. Fibril formation was monitored by ThT fluorescence. Briefly, 2 µL of each sample was mixed with 98 µL of 25 µM ThT in PBS, and fluorescence was measured in a microplate reader using a bottom-read configuration (excitation 450 ± 10 nm, emission 480 ± 10 nm). A pronounced increase in ThT fluorescence, indicative of fibril formation, was observed (Supplementary Fig. 1a). The resulting pre-formed fibrils (PFFs) were aliquoted and stored at − 80 °C.

### Conformational stability immunoassay

The conformational stability of the end products was assessed by an immunoassay. Aliquots (20 µL) were mixed with 20 µL of GdnHCl stock solution to achieve final concentrations ranging from 0 to 6.0 M. After a 1.5-h incubation at room temperature, proteins were precipitated by adding a five-fold volume excess of pre-chilled methanol and storing the samples at − 20 °C overnight. The pellets, collected by centrifugation at 14,000×*g* for 30 min at 4 °C, were re-suspended in 20 µL of classic lysis buffer. Each sample was then digested with proteinase K (PK) (10 µg/mL) for 30 min at 37 °C. The proteolysis was halted by adding complete protease inhibitor cocktail (Roche), after which the samples were denatured and subjected to western blotting on 15% Tris–HCl pre-cast gels (Bio-Rad). The resulting protein bands were analyzed by densitometry, and the GdnHCl concentration required for half-maximal denaturation was determined as the IC_50_.

### Velocity sedimentation in sucrose step gradients

The RT-QuIC end products were resuspended in 20% sarcosyl to a final concentration of 2% and then layered onto 10–60% step sucrose gradients. The gradients were centrifuged at 200,000×*g* for 1 h at 4 °C in an SW55 rotor (Beckman, Brea, California, USA) following a previously described method [28]. After centrifugation, twelve fractions were collected from the top of the gradient and analyzed by western blotting as described below.

### Proteinase K digestion assay

To assess digestion by PK, the end products of the αSyn RT-QuIC assay were subjected to incubation with PK at concentrations ranging from 5 to 50 µg/mL. This incubation was carried out at 37 °C for 30 min under constant shaking at 400 rpm. The proteolytic reaction was terminated by the addition of complete protease inhibitor cocktail (Roche) and then boiled in Laemmli sample buffer supplemented with 10% 2-mercaptoethanol for 10 min, followed by western blot analysis as described below.

### Western blotting

Western blot analysis was performed on αSyn RT-QuIC end products. Proteins were separated by SDS-PAGE using 15% Tris–HCl criterion pre-cast gels (Bio-Rad, Hercules, California, USA) and subsequently transferred to Immobilon-P PVDF membranes (MilliporeSigma, Carlsbad, California, USA) at 70 V for 90 min. The membranes were incubated overnight at 4 °C with the primary antibody, mouse anti-αSyn (10D2), at a 1:4,000 dilution. After washing, membranes were incubated with a horseradish peroxidase-conjugated sheep anti-mouse IgG secondary antibody (1:4,000). Immuno-reactive bands were detected using ECL Plus chemi-luminescent substrate and exposed to Kodak film. Quantitative densitometry of the αSyn bands was carried out using ImageJ software (National Institutes of Health, USA).

### Filtered trap assay

Aggregated αSyn was captured using a filter trap assay, adapted from a previous protocol [28]. In brief, reaction end products (20 µL) were denatured in 150 µL of 2% SDS and applied to a pre-wetted PVDF membrane under vacuum using a Bio-Dot apparatus (Bio-Rad). For immunoblotting, membranes were first probed with a conformation-specific primary antibody (MJFR-14-6-4-2) at a 1:300,000 dilution, and then with a donkey anti-rabbit HRP-conjugated secondary antibody at 1:4,000. Subsequently, to detect the total αSyn content irrespective of conformation, the same membranes were stripped and re-probed with the 10D2 antibody. The assay’s specificity was validated using αSyn monomer and pre-formed fibrils (PFFs) as negative and positive controls, respectively (Supplementary Fig. 1b).

### Transmission electron microscopy

Samples were prepared for transmission electron microscopy (TEM) as previously described [30, 33]. Briefly, 2 µL of each sample was applied to a 200-mesh formvar/carbon-coated grid for 5 min. Excess liquid was gently removed with filter paper, and the grids were negatively stained. Grids were thoroughly examined to qualitatively assess the presence of oligomers or fibrils, and representative images were captured from 15 to 20 distinct locations per grid. Imaging was performed on a JEOL-1400 transmission electron microscope (JEOL USA, Inc., Peabody, Massachusetts, USA) operating at 120 kV. Filament lengths, widths, and volumes were quantified using ImageJ software (National Institutes of Health, USA).

### Statistical analysis

Data are presented as mean ± SEM. Statistical comparisons were performed using an unpaired Student’s t test, one-way ANOVA, or two-way ANOVA, as appropriate, followed by Bonferroni’s post hoc test for multiple comparisons. A *p* value of less than 0.05 was considered statistically significant. Additionally, we used a comparison of receiver operating characteristic (ROC) curves to assess significant differences in the area under the curve (AUC).

## Results

### Comparison of αSyn seeding activity between skin and brain of PD as well as skin αSyn seeding activity among various synucleinopathies

**Fig. 1:**
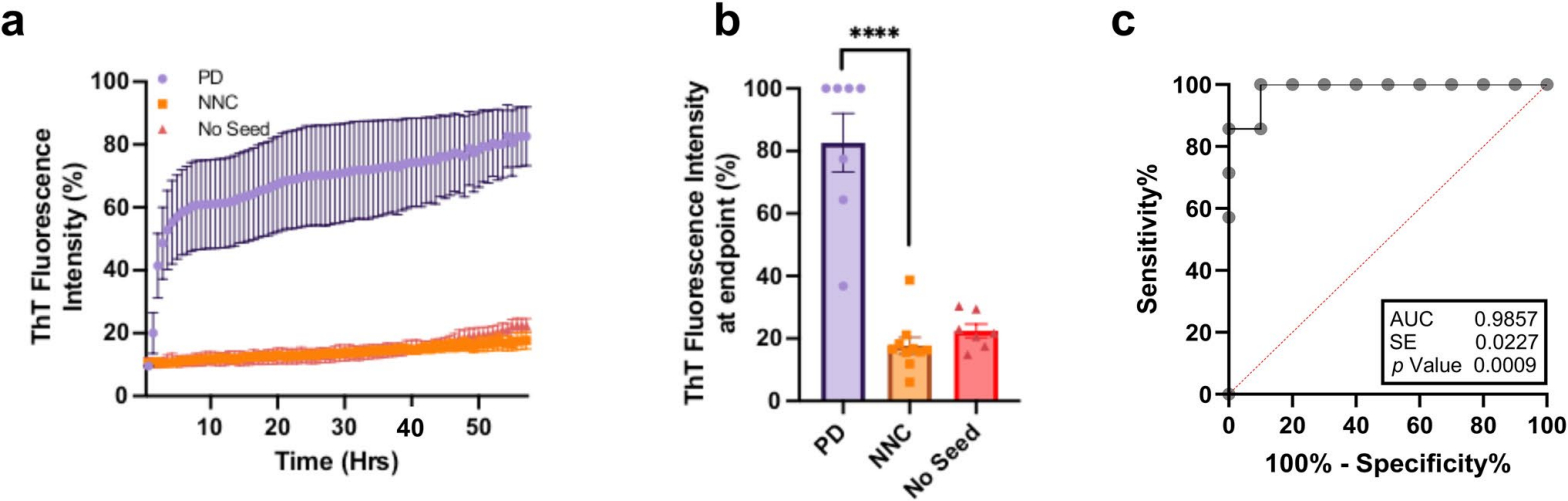
RT-QuIC analysis of seeding activity of brain αSyn from PD and normal controls. **a** Kinetic curves showing the mean ± standard error of fluorescence over time for seed amplification in brain samples from PD (*n* = 7), NNC (*n* = 10), and No seed (*n* = 7). **b** Endpoint fluorescence values of the brain samples analyzed in panel **a**. **c** Receiver operating characteristic (ROC) curve with area under the curve (AUC) for distinguishing PD from NC brain samples based on RT-QuIC seeding activity. ****: *p* < 0.0001

RT-QuIC was employed to assess αSyn seeding activity in brain and skin tissues from individuals diagnosed with PD, DLB, MSA, and neuropathologically normal controls (NNC). Among brain-derived samples, 6 of 7 PD cases (85.7%) tested positive for αSyn seeding activity, with significantly elevated Thioflavin T (ThT) fluorescence intensity compared to controls (Fig. 1a, b). None of the 10 NC brain samples exhibited positive seeding activity, yielding a specificity of 100%, and an AUC of 0.9857 (95% CI 0.9412–1.000; *p* < 0.001) (Fig. 1c).

**Fig. 2:**
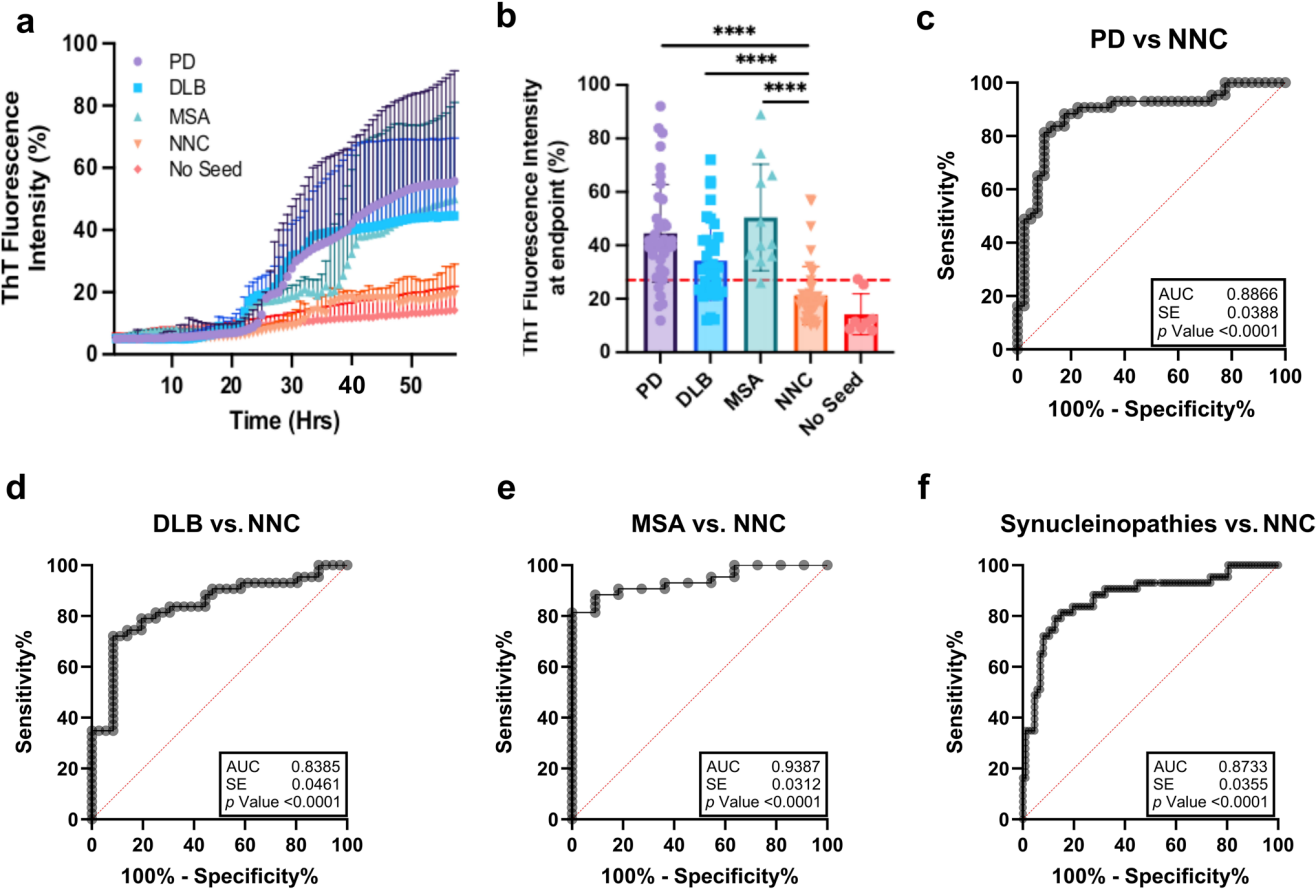
RT-QuIC analysis of skin αSyn-seeding activity from patients with synucleinopathies.** a** Kinetic curves showing the mean ± standard error of fluorescence for αSyn seed amplification of skin samples from PD (*n* = 40), NNC (*n* = 43), DLB (*n* = 36), MSA (*n* = 11), and No seed (*n* = 8). **b** Endpoint fluorescence values for skin samples analyzed in panel **a**. **c**–**f** ROC curves and AUC analyses comparing skin αSyn seeding activity between each synucleinopathy group and NNC controls. ****: *p* < 0.0001

In skin samples, we found an overall increased seeding activities in the synucleinopathies compared to the NNC from the kinetic curves (Fig. 2a) and the endpoint fluorescence readings (Fig. 2b). All the skin samples of patients with various synucleinopathies displayed significantly higher endpoint ThT fluorescence intensity than those of NNC (*p* < 0.0001). ROC curve analysis demonstrated high diagnostic accuracy for skin-based RT-QuIC. The highest AUC was observed for MSA (0.9387, 95% CI 0.8806–1.000; *p* < 0.0001), followed by PD (0.8866, 95% CI 0.8059–0.9609; *p* < 0.0001), all synucleinopathies combined (0.8733, 95% CI 0.7905–0.9333; *p* < 0.0001), and DLB (0.8385, 95% CI 0.7145–0.9135; *p* < 0.0001) (Fig. 2c–f). Non-seeded control reactions (no tissue homogenate) and negative-control samples (NNC) were included in each RT-QuIC plate to verify assay specificity. Both exhibited minimal ThT fluorescence without crossing the positivity threshold, confirming no spontaneous aggregation. Overall, the positive seed-amplification assay (SAA) from the brain samples showed a higher ThT fluorescence intensity and a shorter lag phase than the skin samples at the same tissue homogenate dilution of PD cases. However, no any significant differences in skin αSyn seeding activity among different synucleinopathies were observed based on the RT-QuIC curves (endpoint ThT fluorescence, lag phase, or slope) itself (Fig. 2a, b). Despite the apparent variability in fluorescence kinetics within diagnostic subgroups (notably in PD and DLB, Fig. 2a), post hoc statistical tests revealed that intra-group variation exceeded inter-group differences, highlighting biological heterogeneity across individual cases. This variability was acknowledged as a limitation of the study.

### Proteinase K resistance profiles of αSyn aggregates from RT-QuIC end-product

**Fig. 3:**
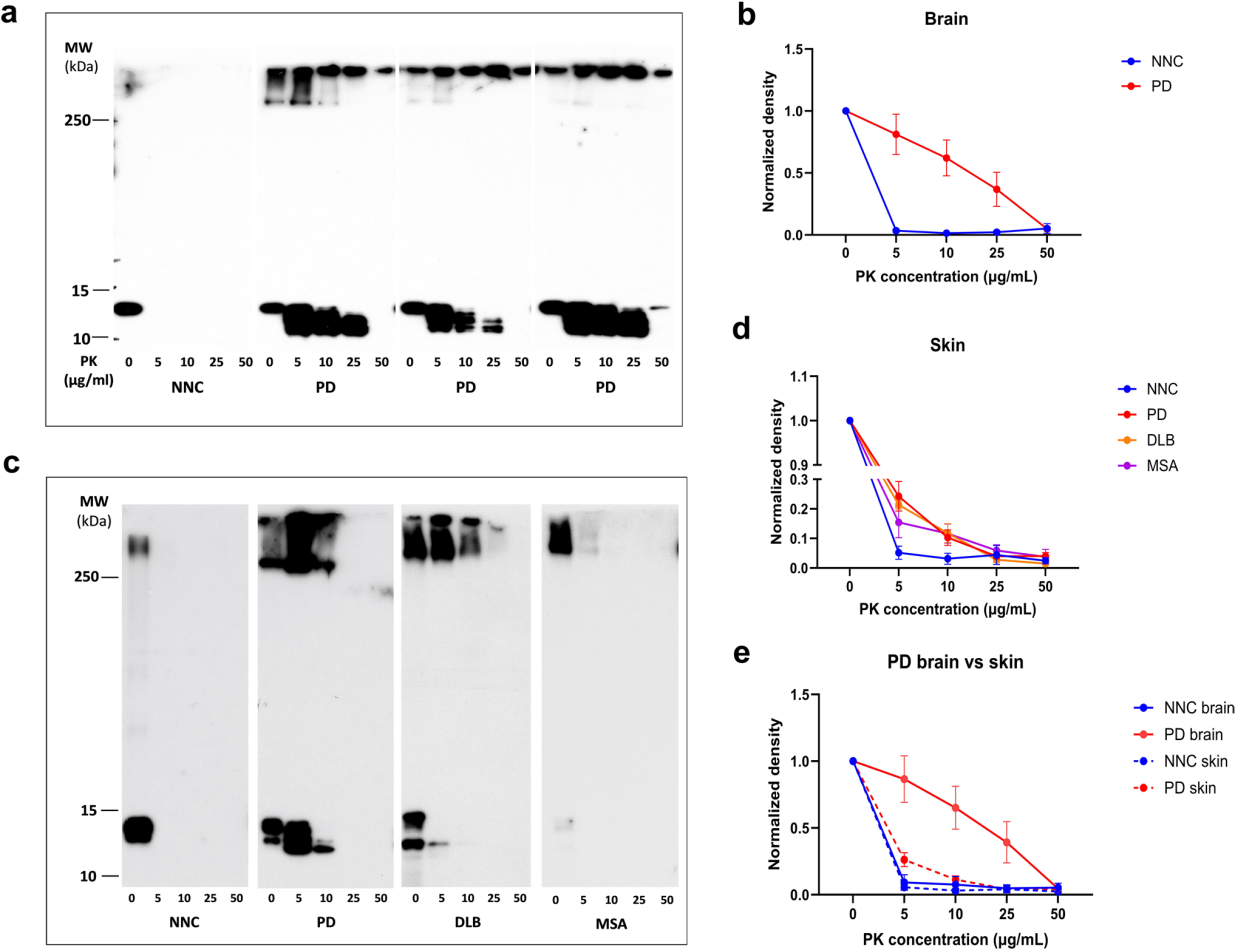
Western blot analysis of different PK resistance of brain and skin αSyn RT-QuIC end products between PD and normal controls.** a** Representative Western blotting RT-QuIC end products of brain αSyn from PD and NNC after treatment with different amounts of PK ranging from 0, 5, 10, 25, and 50 μg/mL. **b** Densitometric quantification assay of Western blotting PK-treated αSyn band intensity from RT-QuIC end products of PD (*n* = 9) and NNC (*n* = 6) brain samples. **c** Representative Western blotting RT-QuIC end products after treatment with different amounts of PK as in a with skin αSyn from PD, NC, DLB and MSA. **d** Quantification of Western blotting PK-treated αSyn band intensity from RT-QuIC end products of PD (*n* = 9), NNC (*n* = 6), DLB (*n* = 4), and MSA (*n* = 4) skin samples. **e** Comparison of quantifications of Western blotting from PD and NNC brain vs. skin

We next performed western blotting on the RT-QuIC end products to examine potential differences in protein band intensity, band ratios, or distribution patterns of amplified αSyn of between brain and skin as well as skin αSyn among different synucleinopathies. RT-QuIC end products (EPs) from brain and skin tissues were subjected to different amounts of proteinase K (PK) digestion to evaluate proteolytic resistance, one of the physicochemical properties of misfolded protein aggregates. αSyn species of brain-derived EPs from PD patients retained PK-resistant at concentrations up to 25 μg/mL, whereas the ones of NNC-derived EPs were fully digested at 5 μg/mL (Fig. 3a). A two-way ANOVA revealed significant main effects of PK concentration [*F*(4, 60) = 21.22, *p* < 0.0001], diagnosis [*F*(1, 60) = 23.80, *p* < 0.0001], and a significant interaction between the two factors [*F*(4, 60) = 4.954, *p* < 0.005]. Post hoc Bonferroni tests confirmed significant differences in αSyn levels between NNC and PD EPs at PK of 5 μg/mL (*p* < 0.0001) and 10 μg/mL (*p* < 0.005) (Fig. 3b).

Skin-derived EPs from PD and DLB samples also exhibited partial resistance at 5–10 μg/mL PK, while MSA-derived aggregates showed reduced resistance (Fig. 3c, d). Two-way ANOVA revealed significant effects of diagnosis [*F*(4, 94) = 917.0, *p* < 0.0001], and PK concentration [F (3, 94) = 4.968, *p* < 0.005] and significant effect of diagnosis interaction [*F*(12, 94) = 2.486, *p* < 0.01], confirming strain-dependent differences in protease resistance. Bonferroni post hoc analysis showed significant differences in protease resistance between NNC and PD EPs (*p* < 0.0001), and between NNC and DLB (*p* < 0.0005) at 5 μg/mL PK. The difference between NNC and MSA did not reach statistical significance (*p* = 0.0626 > 0.05) (Fig. 3d).

Comparison of PK resistance profile of PD skin and brain-derived EPs showed significantly lower protease resistance of skin- than brain-derived ones (Fig. 3e). Two-way ANOVA revealed significant effect of sample origin (skin or brain) [*F*(1, 70) = 26.74, *p* < 0.0001)], significant effect of PK concentration [*F*(4, 70) = 32.18, *p* < 0.0001], and interaction [*F*(4, 70) = 4.920, *p* < 0.005]. Bonferroni post hoc correction revealed significant difference between brain and skin-derived PD EPs at PK of 5 μg/mL *p* < 0.0001), 10 μg/mL (*p* < 0.0005), and 25 μg/mL (*p* < 0.05) (Fig. 3e). Figure 3b, d, and e reflected values normalized using the pre/post-PK WB measurements. These differences suggest that αSyn aggregates amplified from skin may represent an earlier or less compact misfolded strain compared with those derived from brain, possibly reflecting different pathological stages in peripheral versus central tissues.

### Detection of insoluble α-Syn aggregates of brain and skin RT-QuIC end products by filter trap assay

**Fig. 4:**
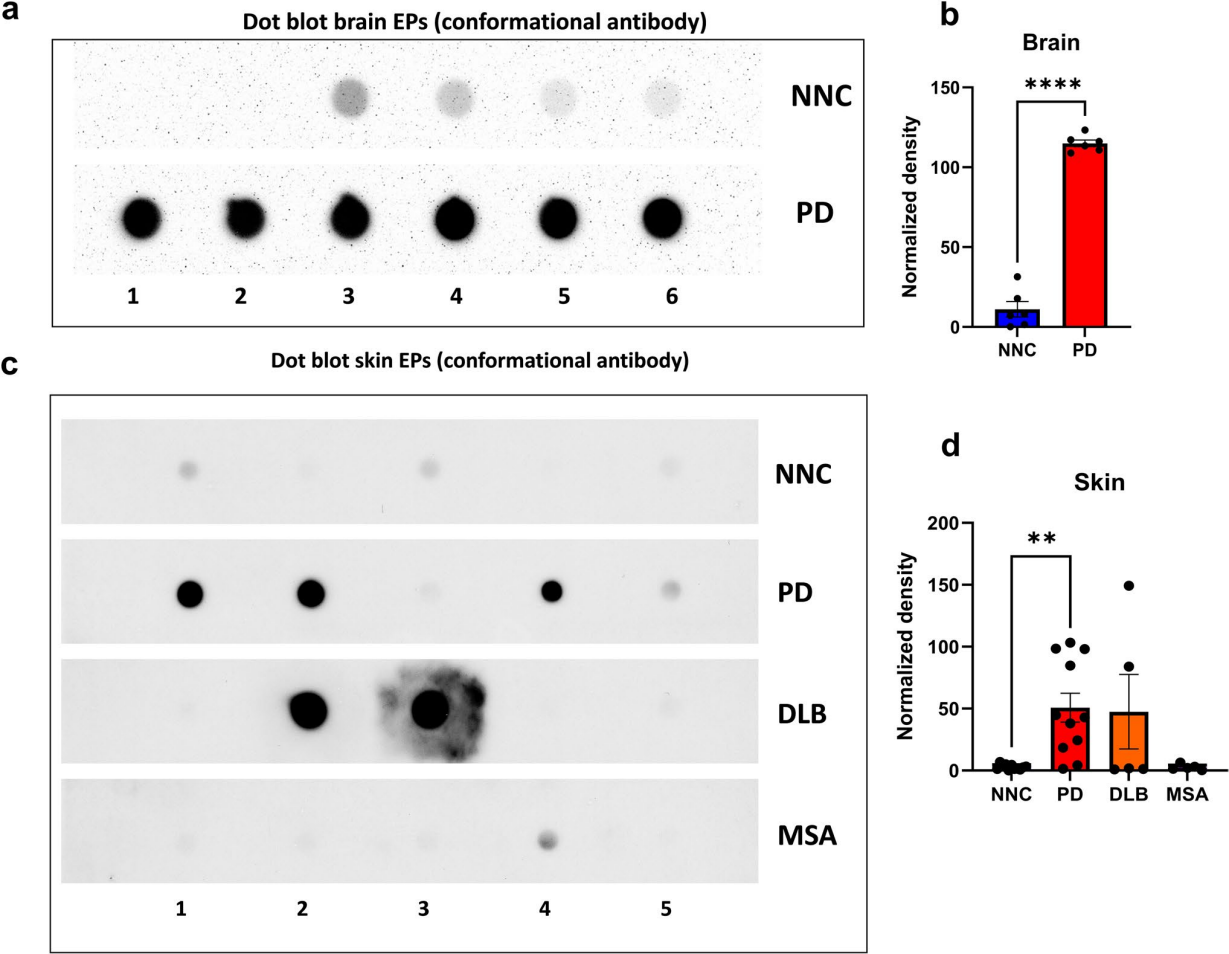
Detection of αSyn aggregates in RT-QuIC end products from various synucleinopathies using filter trap dot blot analysis.** a** and **b** Dot blotting and quantification of α-Syn aggregates of RT-QuIC end products from brain samples of PD (*n* = 6) and NNC (*n* = 6). **c** and **d** Dot blotting and quantification of αSyn aggregates of RT-QuIC end products from skin samples of PD (*n* = 11), NNC (*n* = 11), DLB (*n* = 5), and MSA (*n* = 5). Dot blots were probed with the conformational specific antibody MJFR 14–6-4–2. **: *p* < 0.01; ****: *p* < 0.0001

FTA was used to further determine differences in physicochemical properties of aggregates from brain and skin RT-QuIC end products. PD brain-derived samples showed significantly higher intensity of αSyn aggregates compare to NNC samples (Unpaired *T* test *t*(10) = 19.82, *p* < 0.0001). Among skin samples, DLB and PD displayed substantial aggregate retention, with only PD achieving statistical significance over NNC (One-way ANOVA *F*(3, 28) = 4.974, *p* < 0.01, Bonferroni post hoc correction PD vs NNC, *p* < 0.01). MSA samples showed minimal aggregate retention (Fig. 4c, d). To confirm that differences in MJFR14-6-4-2 immunoreactivity did not simply reflect total αSyn levels, the same membranes were re-probed with 10D2. The aggregate-specific pattern remained unchanged, indicating that filter-trapped signals represented conformationally distinct species rather than abundance differences. The filter trap assay confirmed that only αSyn PFFs were retained on the membrane, whereas monomeric αSyn was not (Supplementary Fig. 1b).

### Comparison of physicochemical properties of αSyn aggregates of RT-QuIC end products by sucrose gradient fractionation analysis

To characterize the solubility and density profiles of αSyn aggregates generated by RT-QuIC, EPs from brain and skin reactions were subjected to sucrose step gradient ultracentrifugation. Fractions were collected from top (low-density) to bottom (high-density) of samples after sucrose density gradient centrifugation and the levels of αSyn in each fraction were analyzed by immunoblotting.

The technique was validated on both the monomeric form of αSyn and PFFs (Supplementary Fig. 2). For all gradient experiments, control gradients using αSyn monomer and PFF standards were analyzed in parallel to define the distribution range of soluble versus aggregated species.

**Fig. 5:**
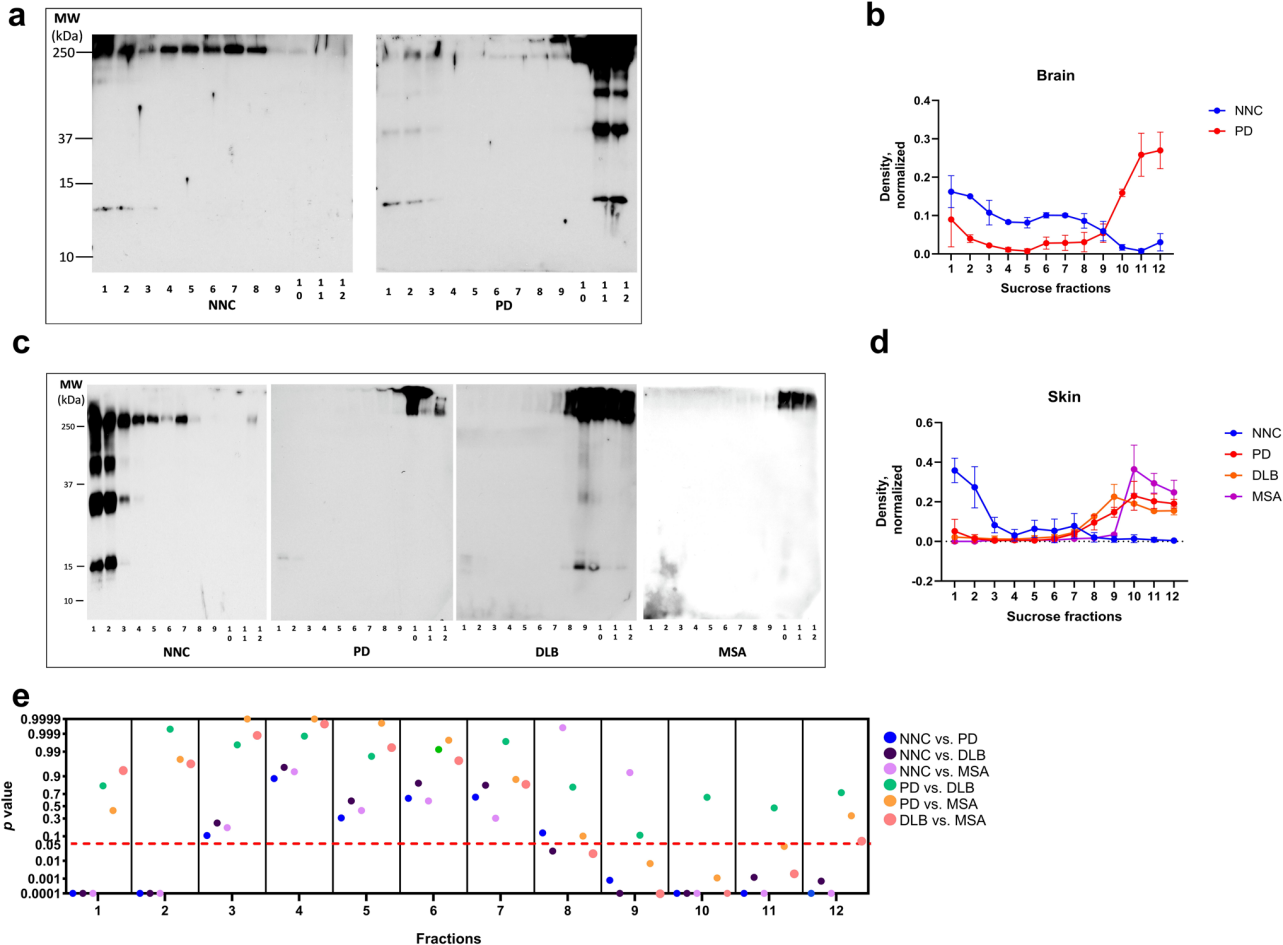
Western blotting of sucrose gradient sedimentation fractions of αSyn RT-QuIC end products of brain and skin from PD and non-PD controls.** a** and **b** Representative Western blotting and quantification of αSyn RT-QuIC end-product of PD (*n* = 3) and non-PD control (NNC) (*n* = 3) brain samples. **c** and **d** Representative Western blotting and quantification of αSyn RT-QuIC end-product of PD (*n* = 3), NNC (*n* = 2), DLB (*n* = 2), and MSA (*n* = 2) skin samples. The blots were probed with anti-αSyn antibody 10D2. **e** Scatter blot shows the *p* value of different groups by *ANOVA.* The red dotted line indicates *p* = 0.05

In RT-QuIC EPs from brain tissues, samples from NNC showed a distribution of αSyn consistent with predominantly soluble forms: monomeric αSyn was concentrated in the top fractions (1–2), while lower-molecular-weight aggregates were detected across fractions 1–8. In contrast, EPs from PD brain samples demonstrated a shift of αSyn toward heavier, less soluble species, with the majority of αSyn aggregates accumulating in the bottom fractions (10–12) (Fig. 5a, b), indicative of higher density and aggregation state.

A similar pattern was observed in skin-derived EPs. NNC samples mostly contained αSyn monomers primarily in fractions 1–2 while small oligomers were observed in fractions 1–7. In contrast, the RT-QuIC end-product samples from patients with synucleinopathies displayed a redistribution of αSyn toward denser fractions: PD EPs showed aggregates concentrated mainly in fractions 9–12, DLB EPs in fractions 7–12, and MSA EPs primarily in fractions 10–12 (Fig. 5c, d). The *p* values from the relevant ANOVA analyses between groups are shown in Fig. 5e, where the red dotted line indicates *p* = 0.05, and all points below this line represent statistically significant differences between the two corresponding groups; fractions 1–12 from the sucrose gradient are arranged sequentially from left to right.

### Conformational stability assay of αSyn from RT-QuIC end products

**Fig. 6:**
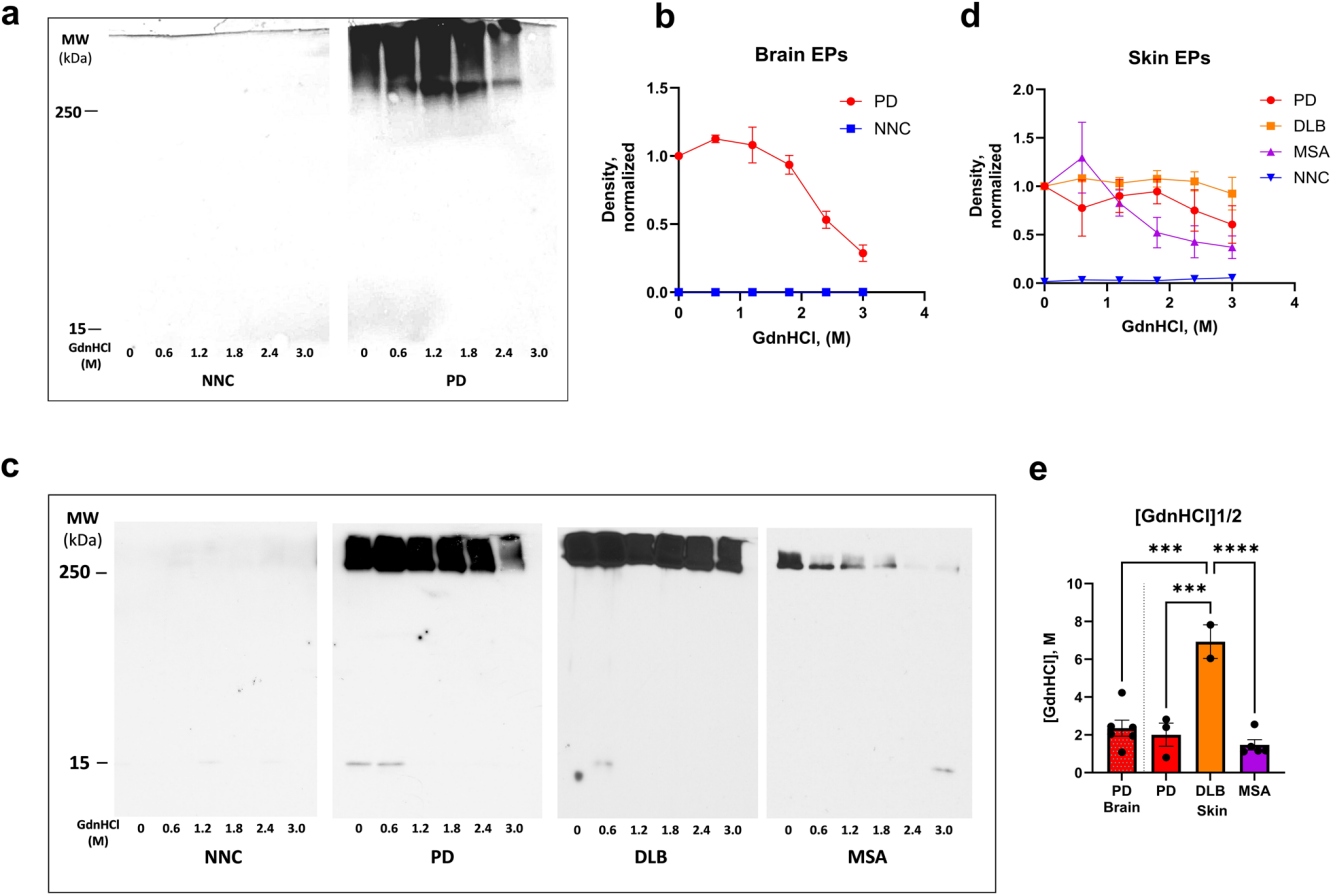
Conformational stability assay of αSyn in RT-QuIC end products across synucleinopathies. **a** GdnHCl titration conformational stability assay of skin EPs from PD and NC samples. **b** Quantification of Western blot (WB) analysis of EPs from PD (*n* = 6) and NNC (*n* = 6) brain samples. GdnHCl titration conformational stability assay of skin EPs from PD, NNC, DLB, and MSA samples. c. and d. Quantification of WB analysis of skin EPs from various synucleinopathies PD (*n* = 3), DLB (*n* = 2), MSA (*n* = 5), and NNC (*n* = 2). e Comparison of [GdnHCl]_1/2 values between brain-derived EPs in PD and skin-derived EPs across synucleinopathies. The blots were probed with anti-αSyn antibody antibody10D2. Statistical significance: ***: *p* < 0.001, ****: *p* < 0.0001

Determining the denaturation effect of different amounts of GdnHCl on proteins or protein aggregates has been often used to reveal differences in conformational stability among disease-specific aggregates [28, 34]. RT-QuIC EPs of both brain and skin samples of NNC exhibited complete susceptibility to proteinase K (PK) digestion, regardless of GdnHCl concentration, indicating the absence of protease-resistant aggregate species (Fig. 6a, b). In contrast, EPs from synucleinopathy samples showed varying degrees of conformational stability as evidenced by differential resistance to GdnHCl-induced denaturation and subsequent PK digestion (Fig. 6a, c, d). The concentration of GdnHCl required to achieve 50% denaturation of αSyn aggregates (GdnHCl1/2) was comparable between PD brain and skin-derived EPs (GdnHCl1/2 PD brain vs. skin: 2.36 ± 0.42 M vs 2.01 ± 0.62 M). Notably, EPs from DLB cases demonstrated significantly greater resistance to GdnHCl (GdnHCl1/2 of 6.93 ± 0.89 M). In contrast, MSA EPs displayed the lowest conformational stability, with a GdnHCl1/2 of 1.47 ± 0.28 M (Fig. 6e). Quantitative values for 50% denaturation (IC₅₀) were derived by fitting the normalized densitometric data to a four-parameter logistic regression. The resulting GdnHCl₁/₂ values indicated that DLB-derived αSyn aggregates exhibited the highest conformational stability, consistent with a more compact, protease-resistant fibril structure. Statistical analysis using one-way ANOVA revealed a significant effect of diagnosis on aggregate stability [*F*(3, 12) = 16.77, *p* < 0.0005]. Bonferroni post hoc comparisons confirmed that αSyn from DLB EPs was significantly more stable than that from PD brain (*p* < 0.0005), PD skin (*p* < 0.001), and MSA (*p* < 0.0001). However, we emphasize that higher aggregate abundance does not necessarily reflect greater conformational stability—these are distinct physicochemical parameters representing quantity versus structural resilience.

### Transmission electron microscopy (TEM) of αSyn from RT-QuIC end products

**Fig. 7:**
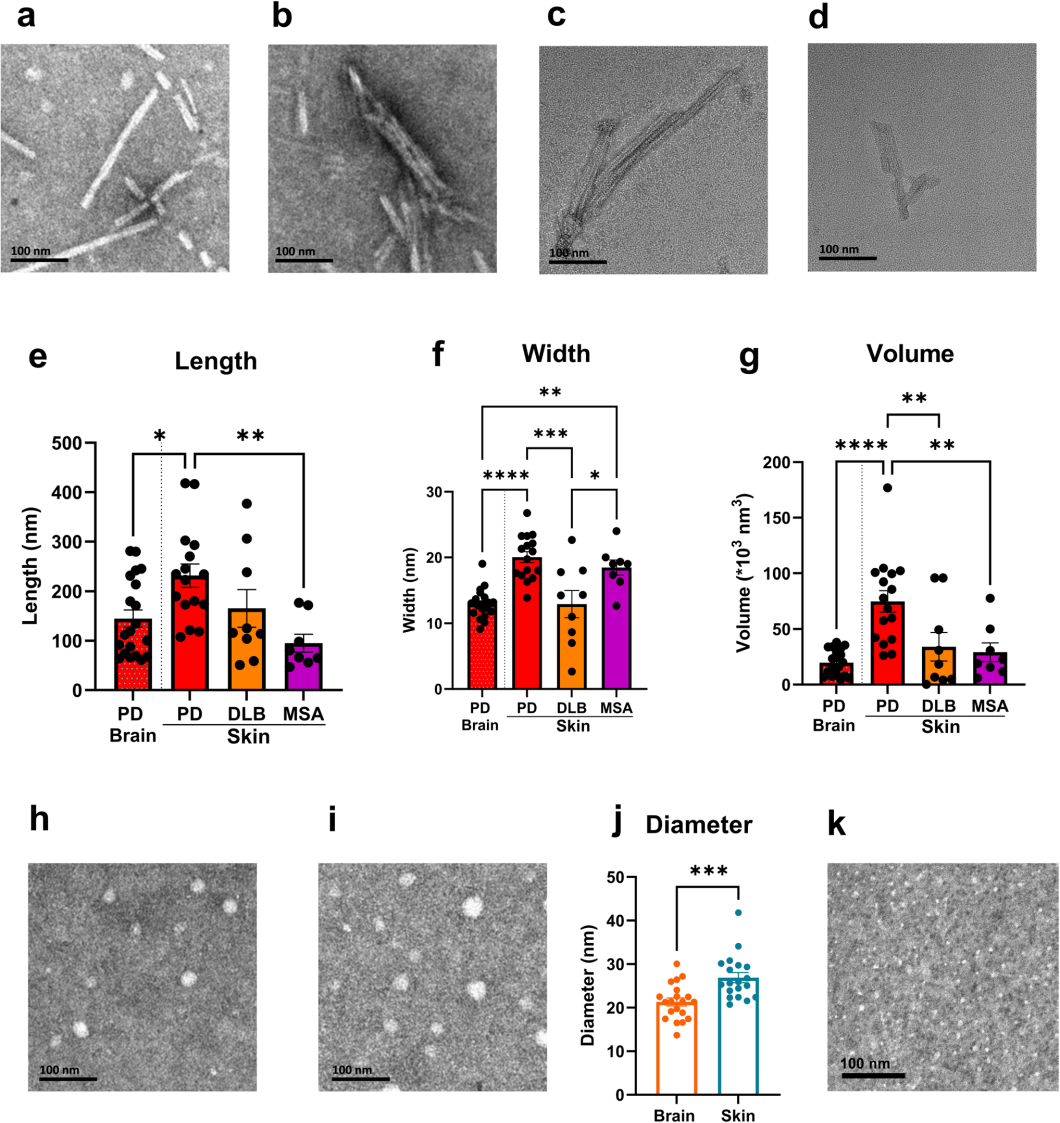
Transmission electron microscopy (TEM) reveals structural differences in αSyn aggregates derived from RT-QuIC end products of brain and skin samples. **a** Representative TEM of amplified αSyn aggregates of PD brain-derived EPs. **b** Representative TEM of amplified αSyn aggregates of PD skin-derived EPs. **c** Representative TEM of amplified αSyn aggregates of DLB skin-derived EPs. **d** Representative TEM image of amplified αSyn aggregates of MSA skin-derived EPs, demonstrating irregular and fragmented fibrils. **e**–**g** Quantitative comparison of αSyn TEM from PD brain and skin RT-QuIC EPs (red bars), and of amplified skin αSyn aggregates from PD and other synucleinopathies (DLB and MSA; orange and purple bars) in terms of fibril length (**e**), width (**f**), and estimated volume (**g**). **h** and **i** TEM images of non-neurodegenerative control (NNC) brain (**h**) and skin (**i**) EPs, showing predominantly spherical structures. **j** Quantitative comparison of the diameters of spherical oligomers in NNC brain vs. skin samples. **: *p* < 0.01, ***: *p* < 0.001, ****: *p* < 0.0001. **k.** Representative TEM of unseeded EPs

A minimum of 10 fibrils per disease group were analyzed. Fibril lengths, widths, and volumes were measured using ImageJ software, and inter-operator variability was < 5%.TEM analysis revealed distinct morphological characteristics of αSyn fibrils formed in EPs seeded with brain versus skin tissue (Fig. 7). Brain-derived EPs predominantly showed well-structured, twisted helical fibrils (Fig. 7a). In contrast, skin-derived EPs exhibited a heterogeneous array of morphologies, including fragmented and irregular fibrillar structures (Fig. 7b–d). Quantitative analyses confirmed significant morphological differences in fibril length [one-way ANOVA, *F*(3, 49) = 5.177, *p* < 0.005], width [*F*(3, 49) = 14.71, *p* < 0.0001], and volume [*F*(3, 49) = 11.59, *p* < 0.0001] among the groups.

Notably, PD skin-derived EPs produced the longest fibrils (231.3 ± 23.36 nm, *n* = 16), significantly surpassing those from PD brain-derived EPs (144.6 ± 17.52 nm, *n* = 20; Bonferroni post hoc comparison, *p* = 0.0230 < 0.05) and MSA skin-derived EPs (95.13 ± 18.15 nm, *n* = 8; *p* = 0.0038 < 0.005), but not significantly different from DLB skin-derived EPs (165.6 ± 38.13 nm, *n* = 9; *p* = 0.5297) (Fig. 7e). The width of fibrils was significantly wider in PD skin-derived EPs (20.05 ± 0.84 nm) and MSA skin-derived EPs (18.46 ± 1.15 nm) than that of fibrils from PD brain-derived EPs (12.96 ± 0.49 nm; PD skin vs. brain, *p* < 0.0001) and DLB skin-derived EPs (12.93 ± 2.07 nm; PD vs. DLB, *p* < 0.005) (Fig. 7f). Additionally, PD skin-derived EPs exhibited notably larger fibril volumes (74,932 ± 9713 × 10^3^ nm^3^) compared to all other groups: PD brain (19,677 ± 2563 × 10^3^ nm^3^, *p* < 0.0001), DLB skin (34,059 ± 12,794 × 10^3^ nm^3^, *p* < 0.01), and MSA skin (29,207 ± 8430 × 103 nm^3^, *p* < 0.005) (Fig. 7g). The variance in fibril volume was also significantly greater in skin-derived EPs—PD skin vs. brain (*F* = 11.49, *p* < 0.0001); DLB skin vs. PD brain (*F* = 11.22, *p* < 0.0001); and MSA skin vs. PD brain (*F* = 4.33, *p* < 0.05)—highlighting enhanced morphological heterogeneity among skin-derived fibrils. Control EPs derived from NNC samples lacked significant fibrillar structures, predominantly forming spherical, oligomer-like assemblies indicative of minimal fibril propagation (Fig. 7h, i). Importantly, spherical particles in skin-derived EPs were significantly larger in diameter than those from brain-derived EPs (26.90 ± 1.17 nm vs. 21.3 ± 0.91 nm; *p* < 0.001) (Fig. 7j). As a control, we also examined the TEM of non-seeded αSyn RT-QuIC end products, which showed no detectable fibrils or proto-fibrils (Fig. 7k). These results collectively indicate that αSyn of skin-derived EPs yielded morphologically diverse and structurally distinct aggregates compared to brain-derived EPs. In addition to fibrillar species, amorphous or spherical particles were detected in NNC samples and interpreted as non-fibrillar oligomers rather than monomers, given that 14 kDa monomeric αSyn cannot be directly visualized under negative staining conditions.

## Discussion

In the present study, we demonstrated significant differences in biochemical, proteolytic, conformational, and ultrastructural properties of αSyn aggregates amplified from skin samples of patients with PD, DLB, and MSA using RT-QuIC assay. Although RT-QuIC has proven effective in differentiating synucleinopathies from non-synucleinopathy controls, our findings indicate that additional characterization of RT-QuIC end products can potentially enable distinction among these clinically overlapping disorders. Our data revealed notable differences in proteolytic resistance to PK digestion among synucleinopathies. Notably, DLB-derived αSyn aggregates exhibited the highest resistance, followed by PD while MSA was the lowest one. Such differential proteolytic profiles might reflect unique structural conformations inherent to αSyn strains in each disorder [23, 31]. In both brain and skin-derived end products, we consistently observed that NNC samples were completely digested at low PK concentrations, whereas samples from synucleinopathy retained high-molecular-weight αSyn species even at higher concentrations of PK. This pattern was most pronounced in DLB, suggesting once again a tighter, more compact aggregate conformation, possibly correlating with slower turnover and increased pathogenic persistence. Using GdnHCl-induced unfolding assays further confirmed substantial variations in conformational stability among RT-QuIC end products. Specifically, DLB-associated aggregates exhibited significantly greater stability compared to those derived from PD and MSA. This observation underscores potential strain-specific differences in hydrogen bonding, β-sheet packing, and tertiary structural rigidity, which may critically influence pathogenicity, transmission properties, and clinical phenotype [1, 5]. The midpoint denaturation values (GdnHCl1/2) showed that DLB aggregates required nearly triple the denaturant concentration to disrupt their structure compared to MSA, suggesting a marked difference in molecular architecture. TEM revealed additional morphological distinctions. Skin-derived aggregates showed increased morphological heterogeneity, featuring longer, wider, and structurally diverse fibrillar assemblies compared to brain-derived aggregates. The average fibril length and width were significantly greater in PD and DLB than in MSA or NNC, and fibril volume variability was also highest in PD. These findings indicate that RT-QuIC can propagate seed-specific morphology, providing an additional phenotypic readout for strain discrimination [2, 21]. Supporting this, filter trap assays showed greater retention of insoluble αSyn aggregates in PD and DLB compared to MSA and NNC in skin-derived EPs. The greater aggregate load in PD samples, confirmed by both endpoint ThT fluorescence and immunoblotting, is consistent with the denser, more stable fibril morphology seen in TEM and the shift to heavier fractions in sucrose gradient ultracentrifugation. In contrast, despite high signal intensity, MSA aggregates had higher solubility and reduced resistance to physical and chemical perturbations.

Interestingly, despite the distinct differences observed among patient-derived αSyn aggregates, artificially aggregated rec-αSyn did not faithfully replicate these unique biochemical and structural features of brain αSyn from patients. This discrepancy suggests that authentic patient-derived seeds, possibly accompanied by unidentified cofactors or post-translational modifications present in skin tissues, are essential for the accurate reproduction of disease-specific strain characteristics. Identifying these factors could illuminate crucial mechanistic pathways in synucleinopathies and potentially uncover novel therapeutic targets [9, 32]. Conceptually, the “body-first vs brain-first” framework provides one explanation for the observed variability: cases with early autonomic involvement may harbor higher peripheral (skin) seeding activity than motor-predominant cases [11, 14]. In addition, lesion “age” (duration from symptom onset) and conformer features (including the flexible “fuzzy coat”) may modulate PK/SDS/GdnHCl profiles—earlier, less compact peripheral aggregates could appear more labile than later, brain-derived species. While our study enhances the current understanding of strain-specific αSyn aggregation properties, several limitations warrant consideration. The sensitivity of RT-QuIC varied significantly between brain and skin samples, suggesting intrinsic differences in seeding potential or accessibility of misfolded αSyn species within peripheral tissues. Moreover, the moderate sensitivity observed in peripheral RT-QuIC assays underscores the necessity of integrating complementary diagnostic modalities to achieve robust differentiation among synucleinopathies in clinical practice. We also note biological and pre-analytical sources of variability: biopsy site/depth, fixation and storage, disease duration, and phenotype (e.g., autonomic-predominant vs motor-predominant). These factors likely contribute to the broader spread of skin-derived kinetic and physicochemical readouts observed here. From a diagnostic standpoint, skin RT-QuIC ROC analyses were reported with stratified fivefold cross-validation and bootstrap 95% CIs, highlighting moderate performance overall and higher AUCs in subgroups with stronger peripheral involvement. Future prospective studies with standardized sampling will be required to define operating points suitable for specific clinical settings.

Despite these strengths, a key limitation of the study lies in the restricted representation of intra-disease heterogeneity and patient-specific variation. The number of analyzed cases per group, although adequate for identifying group-level differences, does not encompass the full clinical and pathological diversity within PD, DLB, and MSA. Variations in disease duration, symptom onset patterns (such as “body-first” versus “brain-first”), treatment history, and comorbidities may substantially influence αSyn aggregate properties. Because all samples were obtained postmortem, temporal aspects of pathology evolution could not be evaluated. Additionally, while we normalized αSyn concentrations to control for abundance differences, residual inter-individual variability may still mask subtle strain or conformational distinctions. Future studies incorporating longitudinal clinical data, stratified sampling by disease subtype and duration, and standardized tissue collection will be essential to fully capture patient-specific and within-disease variability. Another limitation is the reliance on a single amplification condition per tissue type. RT-QuIC kinetics and strain propagation can vary depending on substrate purity, salt concentration, buffer composition, and shaking profile. Although we optimized reaction parameters for reproducibility, alternative conditions could reveal additional strain-dependent behavior. Inclusion of orthogonal amplification systems, such as protein misfolding cyclic amplification (PMCA) or immunocapture-SAA, would help validate whether the biochemical signatures observed here are robust across platforms. Furthermore, while skin provides an accessible peripheral source for αSyn detection, the cellular origin of the aggregated αSyn in dermal homogenates remains unresolved. It is unclear whether these seeds arise predominantly from cutaneous nerve terminals, Schwann cells, or fibroblasts. Future studies using immunohistochemistry, microdissection, or cell type-specific analyses may better clarify the local context of αSyn pathology and its relevance to systemic propagation. Lastly, this study was cross-sectional and performed on postmortem tissues, limiting its ability to establish temporal or causal relationships between peripheral aggregation and disease progression. Longitudinal in vivo biopsy studies, ideally coupled with functional and clinical follow-up, are necessary to determine whether peripheral αSyn seeding activity precedes or reflects central pathology.

In conclusion, our findings elucidate distinct biochemical, structural, and conformational properties of αSyn aggregates generated via RT-QuIC from peripheral tissues, reinforcing the concept of disease-specific αSyn strains in PD, DLB, and MSA. Future investigations focused on identifying cofactors and elucidating mechanisms underlying strain-specific properties hold promise for refining diagnostic criteria and advancing personalized therapeutic strategies in synucleinopathies. Prospective, multi-site studies with standardized skin biopsy protocols (site, depth, processing), pre-registered analyses, and inter-laboratory reference materials will be important next steps. In parallel, correlating skin end-product features with longitudinal clinical trajectories may clarify how peripheral aggregate “age” and conformer stability track disease progression and treatment response.

## Data and materials availability

All materials used in this study will be made available subject to a materials transfer agreement.

## Supplementary Information

Below is the link to the electronic supplementary material.

Supplementary file1 (DOCX 490 KB)

## Data Availability

All materials used in this study will be made available subject to a materials transfer agreement.
