# Supplementary material for: Biochemical signatures of skin α-synuclein in synucleinopathies revealed by RT-QuIC assay end-product analysis
Source: Acta Neuropathol. 2026 Jan 12;151(1):3. doi: 10.1007/s00401-025-02973-5 (PMC12795922; doi:10.1007/s00401-025-02973-5)
Supplement: Supplementary file 1 — Supplementary file1 (DOCX 490 KB) [file 401_2025_2973_MOESM1_ESM.docx]

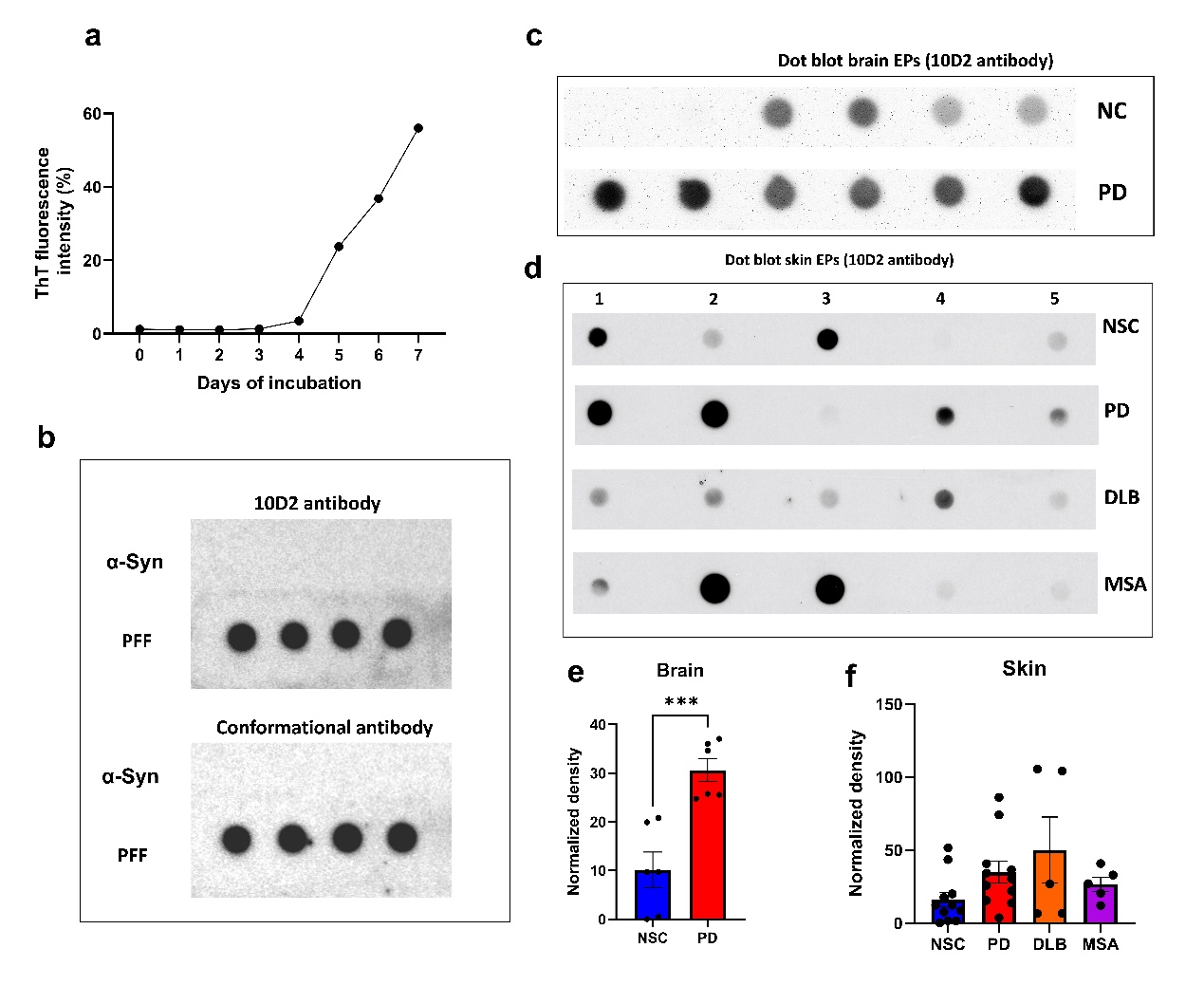


**Supplementary figure 1. Detection of αSyn aggregates in RT-QuIC end-products from various synucleinopathies using filter trap dot blot analysis. a.** ThT fluorescence confirming the PFFs formation. **b**. Filter trap method validation. Dot blot representing the monomeric αSyn and PFFs probed with 10D2 and antiaggregate αSyn antibodies. **c** and **e.** Dot blotting and quantification of α-Syn aggregates of RT-QuIC end-products from brain samples of PD (n = 6) and NNC (n = 6). **c** and **d.** Dot blotting and quantification of αSyn aggregates of RT-QuIC end-products from skin samples of PD (n = 11), NNC (n = 11), DLB (n = 5), and MSA (n = 5). Dot blots were probed with the 10D2 antibodies. ***: *p* < 0.001.


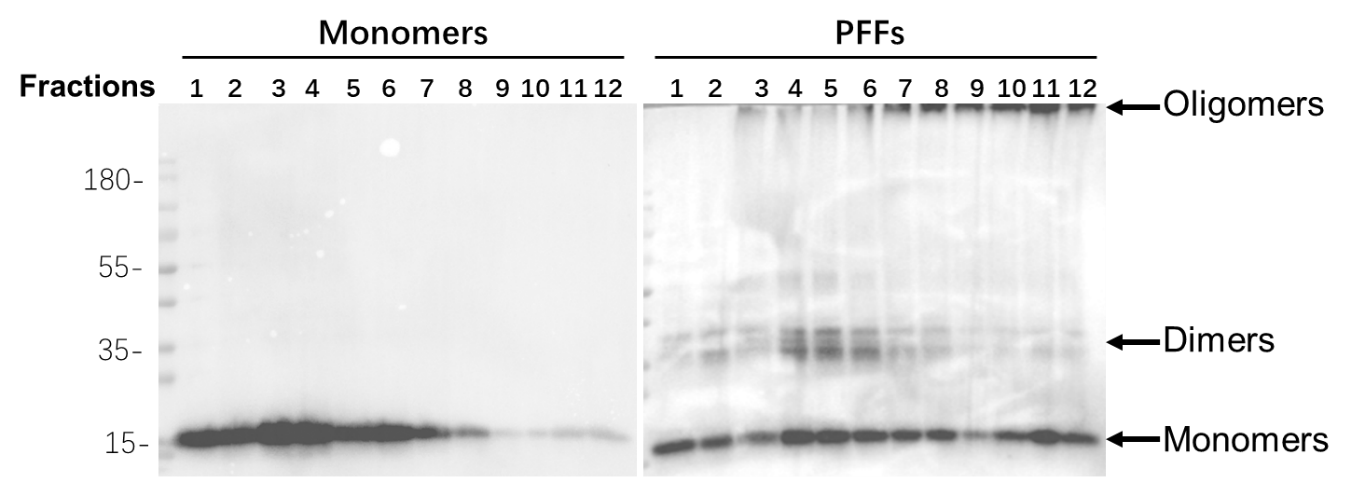


**Supply. Fig 2. Western blotting of sucrose gradient sedimentation fractions of αSyn monomers and PFFs.** Representative Western blotting of αSyn recombinant monomers and PFFs samples. The blots were probed with anti-αSyn antibody MJFR1**.** The membranes were incubated overnight at 4°C with the primary antibody, anti-αsyn antibody (MJFR1), at a 1:4,000 dilution. After washing, membranes were incubated with a horseradish peroxidase-conjugated goat anti-rabbit IgG secondary antibody (1:10000).
